# Molecular perspectives on systemic priming and concomitant immunity in colorectal carcinoma

**DOI:** 10.1186/s43046-024-00211-9

**Published:** 2024-03-11

**Authors:** Suman Kumar Ray, Sukhes Mukherjee

**Affiliations:** 1 Independent Researcher, Bhopal, Madhya Pradesh 462020 India; 2 Department of Biochemistry, All India Institute of Medical Sciences, Bhopal, Madhya Pradesh 462020 India

**Keywords:** Colorectal carcinoma, Immune landscape, Metastasis, Dissemination, Concomitant immunity, Systemic priming, Seed and soil hypothesis

## Abstract

The progression of metastasis, a complex systemic disease, is facilitated by interactions between tumor cells and their isolated microenvironments. Over the past few decades, researchers have investigated the metastatic spread of cancer extensively, identifying multiple stages in the process, such as intravasation, extravasation, tumor latency, and the development of micrometastasis and macrometastasis. The premetastatic niche is established in target organs by the accumulation of aberrant immune cells and extracellular matrix proteins. The “seed and soil” idea, which has become widely known and accepted, is being used to this day to guide cancer studies. Changes in the local and systemic immune systems have a major impact on whether an infection spreads or not. The belief that the immune response may play a role in slowing tumor growth and may be beneficial against the metastatic disease underpins the responsiveness shown in the immunological landscape of metastasis. Various hypotheses on the phylogenesis of metastases have been proposed in the past. The primary tumor’s secreting factors shape the intratumoral microenvironment and the immune landscape, allowing this progress to be made. Therefore, it is evident that among disseminated tumor cells, there are distinct phenotypes that either carry budding for metastasis or have the ability to obtain this potential or in systemic priming through contact with substantial metastatic niches that have implications for medicinal chemistry. Concurrent immunity signals that the main tumor induces an immune response that may not be strong enough to eradicate the tumor. Immunotherapy’s success with some cancer patients shows that it is possible to effectively destroy even advanced-stage tumors by modifying the microenvironment and tumor-immune cell interactions. This review focuses on the metastasome in colorectal carcinoma and the therapeutic implications of site-specific metastasis, systemic priming, tumor spread, and the relationship between the immune system and metastasis.

## Introduction

Cutting-edge technological developments and newer attractive models make a more accessible platform to study metastasis [1]. Approaches like single-cell sequencing of disseminated tumors are very much encouraging to learning metastasome and right opportunities to make some novel idea concerning ideas therapeutic potential [2]. Metastasome is responsible for adhesion, migration, and proliferation. It works as a mediator of intercellular communication [3]. It was a long back to find the history of malignant disease, and literature says it was found in Egypt in 1500 BC [4]. Hippocrates (fifth-century BC) was the first to see the scary lesions in mammary gland carcinoma patients [4]. Despite the several models to find the mechanism of tumor metastasis, it is mysterious and inconclusive clinically [5]. Metastasis is the leading cause of cancer morbidity and mortality, accounting for around 90% of cancer deaths [6]. Even though the cancer survival rate has been meaningfully improved over the years, progress is mainly due to initial diagnosis and malignancy growth inhibition [7]. Partial success has been made in considering cancer metastasis comprising colorectal carcinoma, though existing new-generation anticancer drugs do have effects on metastasis besides their special effects on cancer growth [8]. The cascade includes the development of angiogenesis, detachment, and migration of metastatic cells from the primary tumor, through the membrane and extracellular matrix (ECM) [9]. Then the process continues with the invasion of the membrane supporting endothelium of local blood in addition to lymphatic vessels and then also intravasation of metastatic cells. It is followed by the adhesion of circulating metastatic cells to the endothelium of capillaries of target organs and finally growth of secondary tumors at the target organ site [10]. Researchers have found that metastases, along with metastatically induced cancer cell clone, reveal definite features at the gene, protein, and epigenetic level and in functional level [11]. Physiologically, cells remain within their demarcated periphery through tight cell-cell adhesion and cell-matrix adhesion [10]. Metastatic lesions have less difference with primary tumors, but the acceleration from growth rate in dissemination condition was found in most of the cancers including colorectal carcinoma [12]. Cell adhesion is also important for cancer metastasis, and it is involved in settling metastatic cells at a distal site. It is well known that secondary lesions arise from the seeding and migration of primary tumor cells [13]. Newly, the interplay between primary tumor and secondary organs via circulation has transpired as a crucial factor of tumor metastasis [14]. Neoplastic cells can crosstalk with their surroundings by simple communications, whereas a primary tumor could possibly employ more refined mechanisms to accomplish efficient interactions with distant regions [15]. Antigens should be transported from the tumor site to a lymph node by migratory dendritic cells, and they left the tumor microenvironment or become dysfunctional. If tumor antigen is accepted as “self” during antigen presentation to CD4+T cells, expansion of immunosuppressive regulatory cells can be induced.

### Seed and the soil theory

The “seed and soil” theory of metastasis puts out by Steven Paget was based on a review of 735 fatal breast cancer cases, each having an autopsy and several other cancer cases from the literature. In 1889, the Lancet published his research findings. The “seed” refers to specific tumor cells with the ability to metastasize, and the “soil” is any organ or tissue offering the ideal environment for the growth of the seeds, in his view, such that the distribution of metastases cannot be the result of chance [16]. James Ewing [17] disputed Paget’s “seed and soil” idea in 1928 and proposed that the anatomical makeup of the circulatory system results in pure mechanical processes that cause metastatic dispersion. The idea of “seed and soil” faded into obscurity for the following several decades as a result of not becoming the majority. Hart and Fidler’s groundbreaking from the 1980s confirmed Paget’s “seed and soil” idea by demonstrating the preferred homing of B16 melanoma tumor cells in particularly remote areas. Their research unequivocally established that despite all organs’ vasculatures containing potentially metastatic tumor cells, some organs experienced metastasis development [18].

### Road to study metastasome: from metastasis to metastasis of metastasis

Tumor metastasis is a multistep and multistage process through which malignant cells extend from the primary tumor to intermittent organ sites. Even though tumor metastasis can happen initially in the tumor development period when the primary tumor is small or even undetectable, most happens later when the primary tumor is larger in shape [19]. Researchers have observed that overgrowth and supremacy within primary as well as secondary lesions by a solitary tumor cell population, with identical metastatic signature, are connected with the early metastasis phenomenon [20]. An extension of this hypothesis is that the late appearance of metastatic clones may result in different expression patterns between primary tumors and metastases, secondary to hiding the metastatic signatures in the primary tumors, by continuing non-metastatic clones [21]. On the other hand, investigation of clonal cell lines derived from late-stage carcinomas like colorectal carcinoma has provided direct evidence that discrete cancer cells were coexisting inside a tumor, contrasting in their respective metastatic competence, comprising ones that are non-metastatic, and confirming tumor heterogeneity shown in preclinical investigations with rodent model as well as human tumors [22].

In the year 1889, Stephen Paget, an English surgeon, proposed the “seed and soil” hypothesis. In recent years, more fundamental discoveries have conveyed understanding into our basic understanding of cancer metastasis, and numerous innovative concepts have been recognized [23]. For instance, the “tumor self-seeding hypothesis debated that circulating tumor cells might seed not only to local and distant organs in the body system but also to the original source i.e., primary tumor” [24]. Pre-metastatic niche, hypothesized as a fertile soil favorable to the existence and extension of metastatic seed, has concerned more attention in the period of metastasis research [25]. In past few decades, improvements in technologies like mass spectrometry, microarray, and genome sequencing knowledge have dramatically faster the attempt to widely illustrate the role of metastatic cancer cells and the relationship between primary and secondary tumors [26]. Especially, single-cell sequencing has appeared as a potent technology to characterize the landscape of individual cancer cells like colorectal carcinoma instead of investigating bulk tissue samples composed of millions of cells and has delivered novel insights into our understanding of the multifaceted metastasis systems [27]. Improvement of different microscopic techniques and imaging technology has empowered the visualization and analysis of neoplastic cell dynamics in experimental animals, which may consequently lead to innovative outcomes in metastasis research and promising therapeutic interventions [28].

The generic name for the process by which cancer cells move from the main tumor to nearby tissues and distant organs is metastasis, which accounts for the majority of cancer-related morbidity and mortality [29]. Many sequential and connected processes are involved in metastasis (Table 1). Colon cancer cells must separate from the primary tumor, intravagate to the circulatory and lymphatic systems, avoid the immune response, extravagate at distant capillary beds, and then penetrate and multiply in distant organs in order to complete the metastatic cascade.

**Table 1:** Origin of metastatic colorectal carcinoma cells

Major factors		Description
Seed factors	Epithelial-to-mesenchymal transition (EMT)	• EMT postulates that tumor cells with mesenchymal characteristics develop from either epithelial stem cells or differentiated epithelial cells as a result of a gradual accumulation of gene mutations
	Reentering mesenchymal-epithelial transition (MET)	• In contrast to EMT, MET involves extravasation, invasion, and proliferation at distant sites, followed by the re-expression of epithelial features • EMT is thought to be reversible after the MET • It is unclear how a group of seemingly random somatic mutations could plan the complex set of behaviors associated with the EMT, only for these behaviors to be largely reversed during the MET
	Stem cell origin of metastatic tumor cells	• Populations of tissue stem cells may give rise to metastatic cancer cells • The majority of tissues have cells that are semi-differentiated and capable of replacing dead or damaged cells as a result of normal wear and tear • These undifferentiated or semi-differentiated cells, also known as tissue stem cells, are the source of metastatic malignancies
	Autophagy and metastasis	• Autophagy allows cells to degrade cytoplasmic components in the lysosome, although autophagy has long been hypothesized to play a role in cancer metastasis
	Metastatic dormancy	• Many patients experience a relapse with metastatic cancer months or years after primary tumor treatment due to a clinical phenomenon known as residual tumor cells that can go dormant and grow resistant to treatments • Disseminated tumor cells maintain quiescence, a stable, non-proliferative cellular state, during the period between dissemination and metastatic expansion known as tumor dormancy
	Tumor-secreted extracellular vesicles	• Exosomes, microvesicles, and recently discovered “large oncosomes” are examples of secreted vesicles or extracellular vesicles • Extracellular vesicles are essential in mediating the interaction between tumor cells and host cells, which creates pre-metastatic niche for development of secondary sites
	Tumor-secreted cytokines and chemokines	• Cytokines and chemokines produced by cancer cells have the ability to draw and activate particular cell types • These substances have a variety of purposes, making them important mediators of interactions between cancer cells and their environment
Soil factors	The primary soil factors	• It is well established that the initial tumor microenvironment is essential for controlling cancer spread • The seed-to-soil signaling events that explain how the seed changes the microenvironment have received increased attention in numerous research
	Tumor-associated microphages (TAMs)	• Interleukin-4 (IL-4)-releasing CD4+ T cells trigger the alternatively activated cells known as TAMs
	Mesenchymal stem cells (MSCs)	• It has been demonstrated that mesenchymal stem cells concentrate in breast carcinomas and integrate into the stroma surrounding the tumor • It has been established that MSCs in the tumor stroma increase cancer cells’ capacity for metastasis, which depends on CCL5 signaling through its chemokine receptor CCR5
	Endothelial cells	• Haplo deficiency of PHD2 normalized endothelium lining and vessel maturation, which enhanced tumor perfusion and oxygenation and restricted the capacity of cancer cells to migrate • PHD proteins function as oxygen sensors and may influence oxygen delivery
	Hypoxia in primary soil	• Because tumor cells multiply quickly, the tumor frequently outgrows its blood supply, which causes significant hypoxia • Long-standing research has shown that hypoxia encourages aggressive tumor characteristics, as well as tumor invasion and metastasis • Hypoxia activates Jagged2 in breast cancer cells, initiates EMT, and improves cell survival in vitro, according to research into the molecular mechanism of Notch-ligand activation by hypoxia in primary soil
	The secondary soil factors	• It is possible that “secondary soil” elements, such as the microenvironment of a distant organ or the milieu of a metastatic lesion, play a crucial role in fostering colonization and metastasis expansion • The secondary soil is made up of a variety of elements and cell types that affect the spread of cancer. The pre-metastatic niche has been mostly induced by the intrinsic programs of tumor cells, according to research to date

### The metastatic niche and formation of macro-metastasis in colon cancer

The metastatic niche hypothesis states that while a cell’s precise genetic makeup unquestionably plays a key role in creating its malignant phenotype, microenvironmental conditions are also crucial in allowing malignant cells to reach their potential for metastatic spread. According to the metastatic niche concept, as a tumor moves from a micrometastatic to a macro metastatic state, an appropriately favorable milieu must develop for tumor cells to engraft and multiply at secondary sites [30]. Different models of metastasis phylogenesis have been studied earlier, ranging from the supposition of a common clonal origin for both primary tumor and metastases up to hypotheses of an entirely independent origin of metastasis as well as primary tumor [31]. Allgayer and his group (2020) established strong evidence for a common clonal ancestor of the primary tumor and corresponding metastasis [32]. The hypotheses on an entirely self-governing phylogenesis of a primary tumor and metastasis which is very unlikely at least for that tumor entity [32]. Klein and his group in 2009 worked on a mouse model, and they hypothesized that dissemination of metastatically proficient cells by primary tumor takes place in the initial stages of primary tumor development [33]. They concluded that primary and also secondary lesions of that cancer cells are developing separately.

The linear progression model (type of classical model) accepts metastasis as a chronological event resulting development of primary tumor in one direction [34]. In addition, in one study, Mantovani and his colleagues (2019) detected a tumor protein 53 (TP53) mutation in metastatic condition [35], whereas conforming primary tumor displayed a large deletion spanning TP53 regulator ATM serine/threonine kinase [36].

**Fig. 1:**
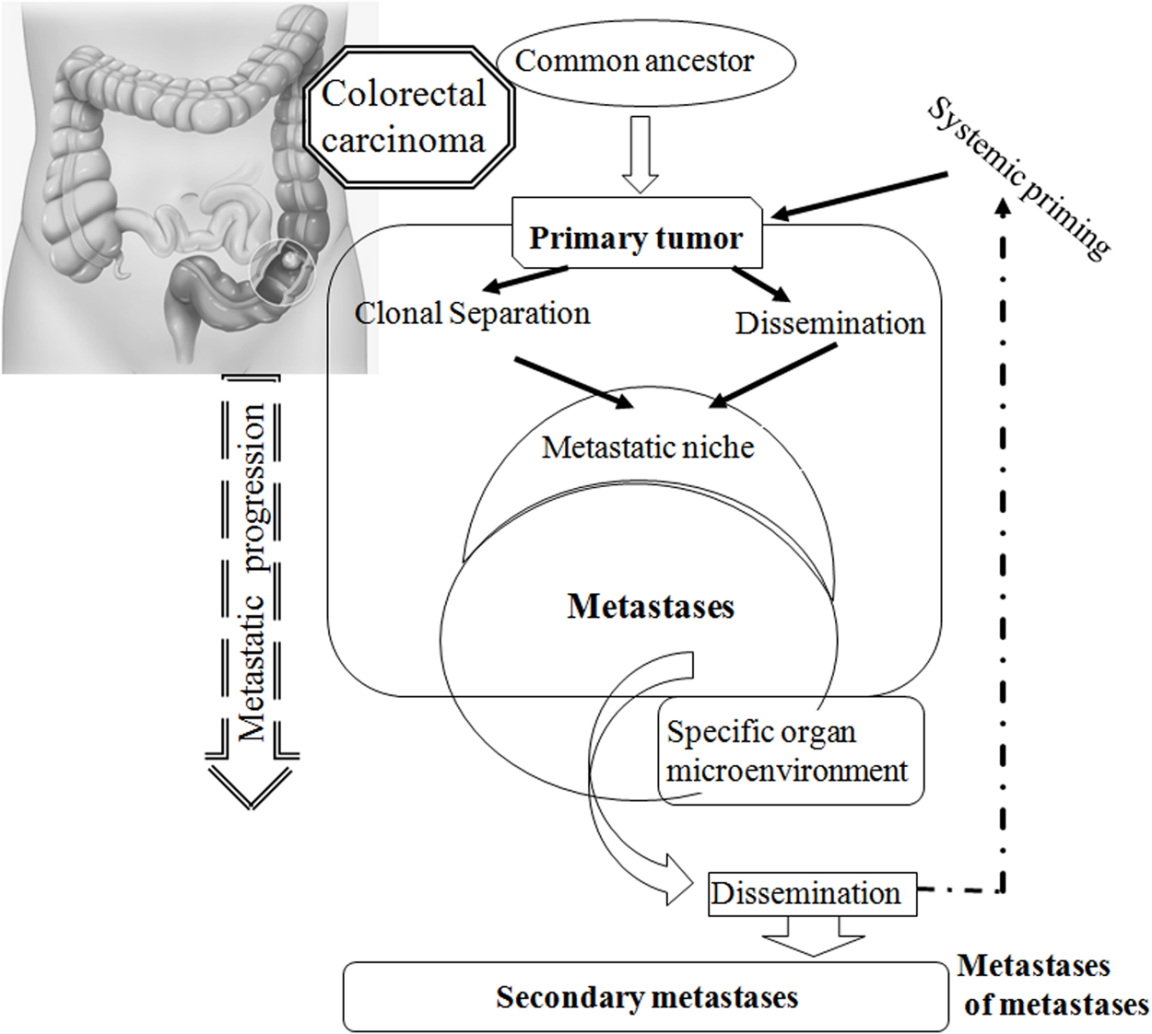
Metastasis of colorectal carcinoma

Since the 1990s, it is known that specific molecules that are detected on some of the disseminated tumor cells for instance urokinase-type plasminogen activator receptor (UPAR) in gastrointestinal carcinoma, epidermal growth factor receptor 2 (EGFR2) in mammary gland carcinoma, etc. associate with later clinical consequence, mainly tumor recurrence as well as metastasis [37]. Thus, it is clear that among disseminated tumor cells, particular phenotypes either already carry the potential for metastasis or have this during the dissemination process [38]. Another metastasis model (also called the self-seeding tumor model), where cancer cells are furthermore extended by metastasis and return to the primary tumor (Fig.1) to contribute to the dynamics of primary tumor genesis [39]. Separation of metastatically relevant clones from their common ancestor in the metastatic process may depend on the timing of specific epigenetic changes as to their consequence for metastatic capabilities [40]. This may be reflect a parallel progression model in cases of prior clonal separation or a linear progression model in cases of later clonal separation [41].

**Fig. 2:**
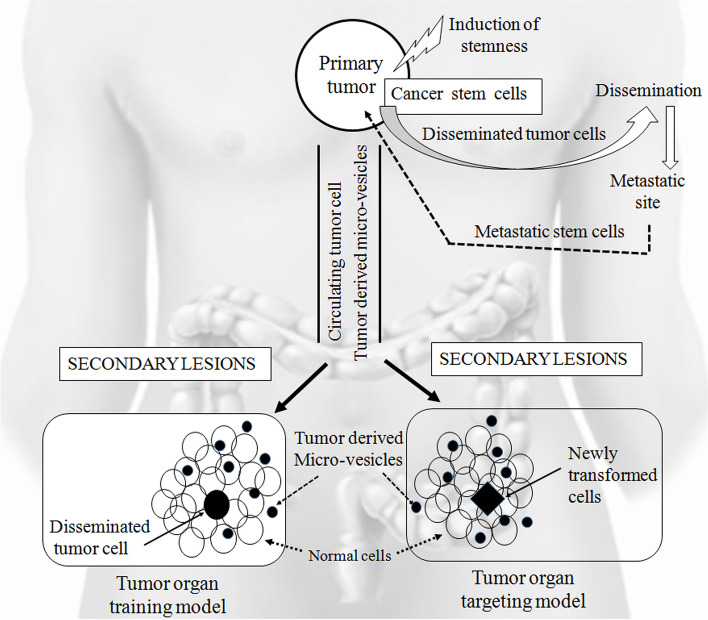
Tumor organ training model and tumor organ targeting models of metastasome and the role of metastatic stem cells and cancer stem cells

Allgayer and his colleagues (2020) analyzed genomic data of 12 colorectal paired metastasis/primary tumor cases, and they found strong evidence that metastasis every time harbors genomic changes that are private to metastasis and not shared with its analogous primary tumor and vice versa [32]. Generally, genomic lesions or metastasis-specific mutations that happened after clonal separation are supported by a single-cell sequencing study of Leung and his colleagues (2017) [42]. Vermaat and his colleagues (2012) conclude a late dissemination model based on their observations in patients with colorectal carcinoma with liver metastases [43]. Based on the literature for colorectal carcinoma, it can be suggested that a common ancestor clone between primary tumor and metastasis, along with initial heterogeneous dissemination that is independent, plays a role in clonal separation [44]. With the concept of metastasome, scientists have suggested two mechanistic models (Fig.2) for tumor metastasis [4], and they are as follows:

- *Tumor organ training (TOTr) model*: Researchers have found that exosomes can induce the expression of a set of metastatic genes in experimental mice models to assist cancer cell recruitment, trapping, and subsequently growth. Furthermore, subsets of metastasis endorsing microvesicles with CSCs property are capable of persuading pre-metastatic niche formation along with metastasis [4].
- *Tumor organ targeting (TOTa) model*: This model fundamentally looks similar to the genometastasis model that states about the cell-free DNAs originated from primary tumors. This cell-free DNA may release into the circulation of oncogenic patients and can be taken up by noncancerous cells in neighboring sites, leading to their transformation as well as the development of second primary tumors. Ghasemi and his groups (2013) proposed a TOTa model which rationalizes the organ tropism of tumor metastasis along with some explanations for morphological similarities between metastases and primary tumors [4].

### Pathogenesis of cancer metastasis: link between concomitant immunity and metastatic cascade

**Fig. 3:**
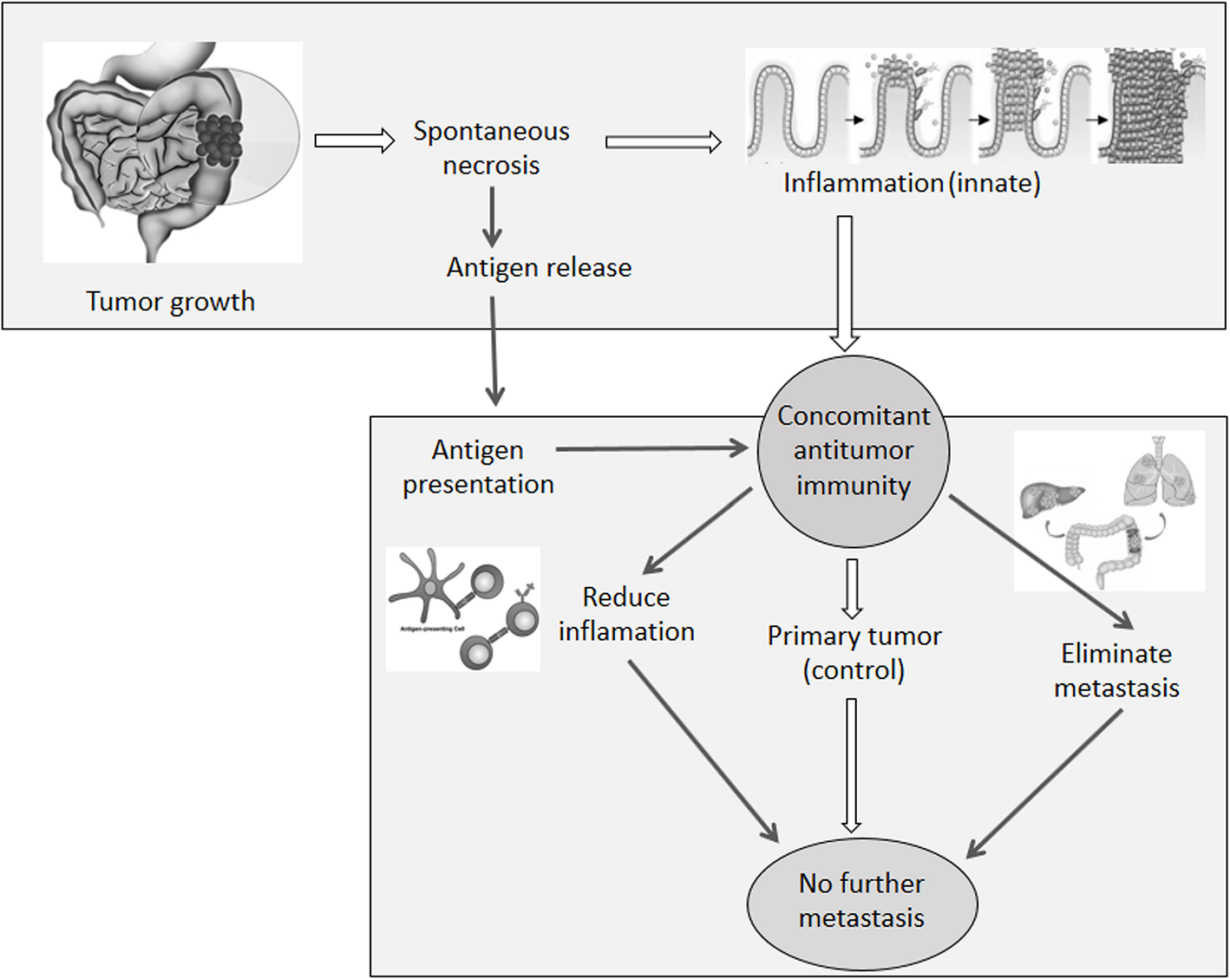
Concomitant immunity and tumor growth

The large list of consecutive, connected processes that make up the cancer-spreading process is extensive. Neoplasms experience a number of alterations over the course of the disease, according to clinical and experimental investigations. One of the major problems to find a therapy for most solid cancers, including colorectal carcinoma, is not the elimination of the primary tumor but rather the eradication of metastases [45]. Metastases upsurge from solitary tumors after neoplastic cells undergo distinctive alterations and headway over a multistep metastatic cascade, forming disseminated tumors which are tough to control. Tumor metastatic progression consists of (a) invasion of neoplastic cells into neighboring tissue at primary tumor site, (b) intravasation of metastatic cells into blood or lymph vessels, (c) survival in circulatory system, (d) extravasation from circulatory system to distant sites, and (e) proliferation in the new environment. Metastasis is a highly complex process [46], and in each step of metastatic cascade, immunogenic cancer cells may be recognized by the host immune system [47]. In addition, under certain conditions, researchers have found that some immune cells in fact favor metastatic disease along with tumor growth [48]. Experimental murine models of metastasis indicated that the advanced growth of a primary tumor inhibited the growth of an experimentally implanted secondary tumor via an immune mechanism, which is referred to as concomitant immunity (CI) (Fig 3) [49].

### Colorectal cancer stem cell and metastasome in immunosuppressive tumor microenvironment

Capability to generate new tumors may be found back to a few numbers of cells within a solid tumor. Cancer stem cell (CSC) hypothesis has been assumed to find the properties of this distinct subset of cells [50]. More investigations of their potential involvement in metastasis resulted in the introduction of a new term, namely metastatic stem cell (metSC) [51]. Remarkably, more investigations publicized that a specific genetic alteration is connected with an amplified expression of CSC-specific transcription factor resembling achaete-scute homolog 2 (ASCL2) in addition to insulin-like growth factor 2 (IGF2) and higher expression of SRY-box 9 (SOX9) and olfactomedin 4 (OLFM4) [52]. They are also identified to be associated in the presence of CSCs in tumors [53].

The presence of metastasis-specific genes or factors like forkhead box protein P2 (FOXP2), protein kinase B (PKB), BRCA2, and casitas B-lineage lymphoma (CBL) is responsible for stemness and self-renewal features of metastasis [54]. MicroRNAs (miRNAs) may play a vital role in the progress of CSCs. Overexpression of miRNA like miR-142-3p leads to loss of breast-CSCs properties supplemented by a reduced expression of CD44 and CD133 and a cell division protein BOD1 (biorientation of chromosomes in cell division protein 1), which are related to cellular stemness [55]. Notch (neurogenic locus notch homolog protein) signaling plays an important role in the existence of CSCs or MetSCs, and it was shown an uncertain performance in the development and progression of various cancers [56] including colorectal carcinoma. Molecular expressions at the functional level of the oncogenetic process will enhance a better idea and clinical relevance of metastasome.

### Metastasis and intratumoral immune cell crosstalk in colorectal carcinoma

Several mechanisms are involved in making an immunosuppressed tumor microenvironment to enable immune escape, thereby motivating tumor progression as well as metastasis formation in colorectal carcinoma. Tumor-derived chemokine like CCL2 recruits monocytes into the tumor microenvironment, and these engaged monocytes can differentiate into tumor-associated macrophages [57]. Regulatory T cells can bind to cytokine IL-2 at a greater affinity than do effector T cells, starving CD8+T cells of IL-2 necessary for CTL activation [58]. Regulatory B cells can produce the inhibitory cytokines IL-10 that may inhibit effector functions of CTLs [59], and it can promote the conversion of CD4+ T cells into regulatory T cells [60]. These mechanisms may contribute to the possibility that tumors can escape immune recognition as well as destruction. CTLs are referred to as the foremost anticancer effector cells [61], and their priming by APCs is the starting phase for effective T-cell reactions. Thus, T-cell priming may be effective along with CTL responses in tumor cells.

### Position-specific metastasis of colorectal carcinoma: ratification from metastasome

Ishaque and his colleagues (2018) have suggested that, during evolution of a metastatic clone that is proficient to colonize a specific organ, also programs for site-specific metastasis [36]. Researchers found a boosted mutational frequency inside PI3K-AKT axis in liver metastases and SRC/PI3K-AKT stimulation for colorectal-liver metastasis [62]. The mechanisms leading to SRC activity during the metastasis process are yet to be completely investigated [63]. In carcinomas, the importance of epithelial-to-mesenchymal transition (EMT) is well documented as an important mechanism to drive metastatic invasion [64]. Concisely, EMT involves in a process of loss of adherents and also tight junctions in epithelial cells which are described by downregulation of E-cadherin expression in addition to the loss in cell polarity [65]. The opposite process of mesenchymal-to-epithelial transition (MET) in contrast to epithelial EMT must be critical features of metSC to propagate and form colonies at target [66]. Ishaque and his group (2018) found that some miRNAs and their regulatory activity make a network of target specifically E-cadherin, SET domain containing 2 (SETD2), forkhead box N3 (FOXN3), zinc finger E-box-binding homeobox transcription factors (ZEB-TF), etc. that are involved in EMT/MET process and thus invasion as well as metastasis [36]. This opinion improves the scenario that EMT/MET organizing processes are crucial for metastasis of colorectal carcinoma to neighboring organs [67].

Vogelstein and his colleagues (1993) postulated a progression model of colorectal carcinoma suggesting consecutive mutational events in diverse signaling pathways [68]. Various studies have shown that alteration of signaling pathways like Wnt, TP53, and TGFβ is related to progression and metastasis of colorectal carcinoma [69]. A whole-genome study was done by Allgayer and his group (2020) to compare primary tumors with corresponding metastases form of colorectal carcinoma, and they confirmed the “Vogelstein sequence” [39]. Furthermore, interesting noncoding mutational events may enhance the progression model and ought to be explored functionally in future investigations [70]. Binnewies and his colleagues (2018) have found that metastatic progression, as well as metastatic switch, is associated with alterations of tumor immune microenvironment (TIME) performed in modulation of immune evasion [71].

Müller and his group (2001) have shown that chemokine induces rearrangement of cytoskeleton, adhesion of integrin, directional migration, etc. that are vital for localization of metastatic cancer cells to selective sites [72]. A number of researchers have reported the role of chemokine receptor CXCR4 and its ligand CXCL12 in metastasis [73] in many cancers including colorectal carcinoma.

### Metastasome and its clinical implications

A recent whole-genome analysis study was conducted by Ishaque and his colleagues (2018) who found an indication that the metastases harbor genomic changes and are not being shared with the primary tumor [36]. Another methodological explanation may be that cancer cell population is giving rise to metastasis that is present in analogous primary tumor; however, in cells, numbers are too small to detect using the genetic signature of other subclones. This undoubtedly may be one clarification for an unaffected situation that metastases still arise afterward total therapeutic removal of primary tumor which causes maximum deaths related to cancer including colorectal carcinoma [74]. Different approaches of tumor cell migration that may permit therapeutic consequences at the individual level and initiatives must be propelled by combining imaging (micro with macroscopic level) synergistically [75].

Innovative molecular tracer’s techniques are used to visualize genomic changes in metastasis conditions, and this can be presented in the oncology department for the diagnostic purposes [76]. Recently, innovative biomarkers were found in primary tumors as well as in metastasis utilizing for a diagnosis like EGFR-based therapies, namely cetuximab [77]. They found that metastasis special mutations occur within FAT1, which modulates EMT and metastatic stemness and also induces unusual Wnt signaling during colorectal carcinoma, and these observations have the potential to make a strategy for targeted treatment [78]. Vermaat and his colleagues (2012) studied the role of mutated kinase insert domain receptor (KDR) and vascular endothelial growth factor receptor 1 (VEGFR1) gene which are related to colorectal carcinoma metastases and might have therapeutic potential [43]. Scientists have studied SMAD2 and SMAD4 proteins during colorectal carcinoma progression and genomic alterations within TGFβ pathway to find a new therapeutic strategy [79]. SRC inhibitors like dasatinib are now being used as a tool against liver metastases of colorectal carcinoma, after proper understanding of the role of SRC and its activation during metastasis of colorectal carcinoma [80].

### Concomitant immunity as a therapeutic target to prevent metastasis

Concomitant immunity is the occurrence of secondary tumor rejection during primary tumor growth, seen in many experimental animal models of malignancy. It can be incited by numerous tumor-determined stimuli, and diverse immune cell subsets may either promote or hinder metastasis. Important actors are T cells, NK cells, and M1-like macrophages that can perceive and slaughter metastatic cancer cells in addition to Tregs and M2-like macrophages that are modified by the tumor to go around concomitant immunity over inhibition of T cells and NK cells. Different studies exhibit how inhibition of explicit concomitant immunity mechanisms quickens metastatic growth. Consequently, expanded comprehension of concomitant immunity may give a few new focuses to cancer treatment. Concomitant immunity appears to often weaken as time advances, and metastasis happens [49]. Hindrance of prostaglandin E2 synthesis reestablished the anti-metastatic impact of late concomitant immunity macrophages [49]. This model highlights the significance of new investigations, as they legitimately recommend explicit mediations to support concomitant immunity against metastasis. For instance, explicit hindrance or depletion of Tregs would fortify cytotoxic CD8+ T cell and NK cell function in both primary tumors and in circulation. This may prevent the early dissemination of malignancy cells from the primary tumor while expanding anti-tumor impact against already disseminated tumor cells in circulation or recently seeded tumor cells at other sites [81]. Remarkably, ongoing proof recommends that isoform-explicit hindrance of PI3K-Akt pathway specially inhibits Tregs with insignificant impact on concomitant immunity as a therapeutic target to forestall metastasis.

### Metastasis as a systemic disease: molecular insights and clinical implications

The main factor contributing to cancer-related death is metastasis. Since primary tumors can change their local and systemic surroundings in ways that promote metastasis development, recent results from experimental models and clinical experience imply that cancer is a systemic disease even at early stages [82]. Even in individuals with early-stage disease, disseminated and circulating tumor cells can be found [83]; while not all of these cells progress into full-blown metastatic lesions, their presence signals metastatic spread and supports clinical prognostication. Numerous tumor-derived substances affect distant organs systemically and activate local inflammatory and mesenchymal cells to generate pre-metastatic and post-dissemination niches that promote metastatic expansion. In keeping with this, immunotherapy [84, 85] is one of the few curative treatments even for diffusely metastatic illness; however, it has only been successful in treating a small subset of patients and certain cancer types. The development of novel therapeutic strategies [86] focused on treating disseminated disease should be made easier with a better understanding of tumor-induced and host-related systemic implications on metastasis.

## Conclusion and future aspect

Malignancy can be extended throughout the body of malignant patients via circulating tumor-derived microvesicles, but cell migration is a vital phenomenon that must be accomplished for fulfillment of the progression. Predominantly, the association and structure of cells basically malformed in the time of neoplastic development, and cancer is a disease of a precise tissue, not cell, thus gaining immense clinical relevance. An innovative concept like metastasome and its expanded information can generate novel concepts into the existing body of information to make a novel therapeutic approach in the field of oncology and will be a useful tool for clinicians in dealing with the scenario.

Upcoming therapies for metastatic colorectal carcinoma would perhaps be oriented to target the previously disseminated tumor cells in addition to damaging their growth and development into clinically relevant metastases since this is the controlling step in metastatization for most carcinomas. In this aspect, it will be vital to find an extensive genomic as well as molecular characterization of metastases related to their primary tumors’ active epigenetic drivers of metastasis. Metastasome is an emerging field with great promise, but until today, insufficient research work has been done using its noble concept. We hope the study of metastasome in addition to novel-targeted therapeutic approaches will be helpful to cancer patients in the modern era of precision medicine.

Not only primary tumor but also furthermore plastic nature of individual immune cells as well as functions can move the tumor immune microenvironment in the direction of an immunosuppressive, pro-tumor environment, debilitating concomitant immunity and empowering immune escape. Furthermore, the naturally happening metastatic process of tumor cell separation from the primary tumor, intravasation, endurance in circulation, and extravasation into target tissue are all not reiterated in models where a direct infusion of secondary tumor cell inoculum recreates metastasis. Taking everything into account, concomitant immunity plays a significant and varied role in all steps of the metastatic cascade. Numerous particular targets in the association cons associated with concomitant immunity and metastatic cascade have been recognized, taking into account the rational design of interventions that fortify the concomitant immunity to forestall metastasis and in this way diminish cancer morbidity as well as mortality.

## Data Availability

Data of the review are publicly published data. The study does not require ethical approval.

## Abbreviations

BC: Before Christ
ECM: Extracellular matrix
TP53: Tumor protein 53
ATM: Ataxia-telangiectasia mutated
TOTr: Tumor organ training
TOTa: Tumor organ targeting
CSC: Colorectal carcinoma
Metsc: Metastatic stem cell
ASCL2: Achaete-scute homolog 2
IGF2: Insulin-like growth factor 2
SOX9: SRY-box 9
OLFM4: Olfactomedin 4
UTR: Untranslated region
FOXP2: Forkhead box protein P2
PKB: Protein kinase B
BRCA: BReastCAncer gene
CBL: Casitas B-lineage lymphoma
MiRNAs: MicroRNAs
BOD1: Biorientation of chromosomes in cell division protein 1
NOTCH: Neurogenic locus notch homolog protein
PI3K-AKT: Phosphatidylinositol 3-kinase/protein kinase B
SRC: SRC proto-oncogene, non-receptor tyrosine kinase
EMT: Epithelial-to-mesenchymal transition
MET: Mesenchymal-to-epithelial transition
ZEB-TF: Zinc finger E-box-binding homeobox-transcription factors
FOXN3: Forkhead box N3
SETD2: SET domain containing 2
EGFR: Epidermal growth factor receptor
FAT: FAT atypical cadherin 1
KDR: Kinase insert domain receptor
TGFβ: Transforming growth factor beta
mTOR: Mammalian target of rapamycin
